# Marked distinctions in syntactic complexity: A case of second language university learners’ and native speakers’ syntactic constructions

**DOI:** 10.3389/fpsyg.2022.1048286

**Published:** 2022-11-25

**Authors:** Jing Lyu, Muhammad Ilyas Chishti, Zhibin Peng

**Affiliations:** ^1^Academy of Global Governance and Area Studies, Sichuan Normal University, Chengdu, Sichuan, China; ^2^National University of Sciences and Technology (NUST), Islamabad, Pakistan; ^3^Foreign Language Research Department, Beijing Foreign Studies University, Beijing, China

**Keywords:** syntactic complexity, L2 proficiency, L2 development, genre, academic writing

## Abstract

Existing research on syntactic complexity tends to examine diversity and complexity embedded in grammatical employments which may well be witnessed in formulations of different syntactic structures. Conceivably, the subject syntactic complexity seems to be exercised mostly by non-native speakers and writers. The present study employs pseudo-longitudinal data: essays written by undergraduate students in different levels collected at the same time. Hence, it aims at investigating the mechanism involved in the L2 production of L2 learners in terms of syntactic complexity by analyzing a corpus of non-native English-speaking learners. The research inquiry is mainly focused on investigation of significant differences in terms of syntactic complexity between writing of Japanese university students and that of native speakers, probing further into the particular dimension and level of difference. The study also traces marked variations in syntactic complexity employed by Japanese university students in different grades. Nagoya Interlanguage Corpus of English (NICE) developed by Sugiura in 2015 was employed to conduct the entire research proceedings. The corpus of the subject study comprises 339 essays written by L2 EFL learners studying in a Japanese university employing a judicious selection of quantitative measures of syntactic complexity. The results exhibited a considerable statistical difference between the writing of Japanese learners and native English writers. The findings of this study provide meaningful pedagogical implications for English teachers and textbook compilers. Japanese university students in higher grades are found to be employing more complicated and diverse syntactic structures. Conforming to the already conducted research studies with almost similar objectives, this study demonstrates the significance of using both general and more particular complexity metrics to assess syntactic development in L2.

## Introduction

Writing has often been considered the most challenging skill to acquire even in L1. However, the task gets doubly challenging when it comes to skill development in academic writing in second language acquisition. In this very connection, writing competence has always remained a widely debated and discussed issue within academic settings which gained special attention in applied linguistics. L2 writing performance and development from multiple angles and within various contextual settings have been the areas of research during the last couple of decades. Out of various levels of proficiency, linguistic and syntactic complexity gained more prominence within academic research. Syntactic complexity may be viewed as the variety and intricacies of grammatical resources that are exhibited in the creation of a language in general terms. All modern definitions of high currency among second language (L2) scholars encompass synonyms and other features including variety, diversity, and elaborateness of deployed grammatical elements which are often encountered. As a dependent variable, it is generally examined in terms of the quality of language output that is predicted to consistently fluctuate in response to various external factors (Ortega, 2003).

Afzaal et al. (2021) pinpointing the dimensions of coherence in academic writing suggest that English has turned out to be a dominant language in academic research in writing. Adding, they remark that competence to write not only coherently but also cohesively in English has become a compulsion in academia today. Mohan and Lo (1985) (as cited in Afzaal et al., 2021), make it more explicit stating that students employ a bunch of linguistic markers augmenting the voice of writers within their work, e.g., native writers follow conventions of academic writing instinctively, for instance, incorporation of clarity, discourse markers, hedges and other cohesive transitions. However, within the context of syntactic complexity, a large set of linguistics features, such as length of syntactic unit, amount of embedding, range of structural types, and sophistication of structures, are used to gauge the degree of syntactic complexity. Many researchers have attempted to investigate syntactic complexity in one way or another, as the growth of syntactic competence has been considered essential to an overall development in the target language (Ai and Lu, 2013). Unfortunately, due to the lack of reliable computation system for syntactic complexity analysis, most of the previously conducted studies owe to very few measures and relatively small amount of data.

As per the previously conducted research (Biber and Clark, 2002; Ortega, 2003; Bell, 2007; Daller and Xue, 2007; Biber and Conrad, 2009; Bjork et al., 2009; Adel et al., 2012; Bjorkman, 2013; Lu, 2017; Bulté and Housen, 2018; Kyle and Crossley, 2018; Liu and Afzaal, 2021), any increases in syntactic complexity that are observed—whether cross-sectionally or longitudinally—must be seen as a reflection of the interplay of the following elements at the very least: taught development, first language, and mode of communication. Any of these elements might be considered independent variables in and of itself, deserving further investigation. Any of these dimensions can be viewed as a moderating factor at various points throughout time. Recent corpus-based L2 writing studies have increased our understanding of the link between syntactic complexity and quality of writing in L2, as well as the influence of other task factors on this relationship; thanks to the development of computational tools for syntactic complexity analysis. This on-going line of study has provided valuable first insights into how to improve the operationalizations of syntactic complexity in L2 writing evaluation by discovering additional sources of information. Ortega (2003) reviewed 25 studies which all attempted to measure learners’ language development and found that those studies only used three kinds of syntactic complexity measures in average and examined samples whose total numbers range from 16 to 300. Bulté and Housen (2018) are of the view that second language development, and particularly L2 complexity growth, is viewed as a dynamic process that can progress gradually or abruptly, but can also be marked by phases of backsliding and stasis. Even while broad developmental patterns and trends may arise among learners (i.e., in groups of learners), there is no such thing as “the typical learner,” and the developmental routes of individual learners must be researched to have a comprehensive understanding of the developmental process.

However, within second language acquisition, linguistic (or structural) complexity and its subcomponent syntactic complexity have been investigated for a variety of reasons and from a variety of theoretical perspectives, employing a variety of methodological approaches (Ortega, 2003; Bulté and Housen, 2018).

Most of the studies that have delved deeper into the development of L2 complexity and its relationship with overall L2 proficiency, and L2 development are witnessed to be entirely cross-sectional in nature and have not examined the actual development of individual learners with a passage of time. Only a very small number of studies have researched into longitudinal development; either encompassed a relatively short span of time, taken into account only very few data collection points, or comprised only a small number of learners (Larsen-Freeman, 2006; Verspoor et al., 2008; Byrnes et al., 2010; Spoelman and Verspoor, 2010; Polat and Kim, 2014).

### Syntactic complexity in academic writing

The topic of complexity in language has been investigated and addressed from several angles in previous studies such as Milroy and Milroy (1985), Mauranen (2003, 2009, 2012, 2017), Ortega (2003), Norris and Ortega (2009), Ranta (2013), Pallotti (2014), McCambridge (2015), Mur-Duenas (2015), Politzer-Ahles et al. (2016), and Bulté and Housen (2018). For example, in SLA research, syntactic complexity is employed as an indicator of learners’ language proficiency (Crossley and McNamara, 2014), to measure language proficiency (Ferris, 1994; Ortega, 2003), and to test the effectiveness of specific pedagogical interventions (Wolfe-Quintero et al.,1998; Ellis and Ferreira-Junior, 2009; Ong and Zhang, 2010; Crossley and McNamara, 2014). These studies highlighted that the syntactic complexity is based on “looking at the number of linked elements in a structure, and length of the sentences.” These linked components can be phrase length, phrase number per clause, and clause number per unit (Neary-Sundquist, 2017).

The pertinent interest of the previous studies was on the difference in syntactic complexity between native speakers (NS) and non-native speakers (NNS) in terms of length of production unit, amount of subordination, amount of coordination, and degree of phrasal complexity (Swales, 1990; Seidlhofer, 2004, 2005, 2011; Foster and Tavakoli, 2009; Seidlhofer and Widdowson, 2009; Römer and Wulff, 2010; Tang, 2012; Ai and Lu, 2013; Mancilla et al., 2017). However, due to genre variations, the findings of these research studies have not been consistent. NNS utilize more coordination and complicated words but less subordination than NS in online conversations, however high level NNS writing approaches NS writing in terms of subordination (Mancilla et al., 2017). NNS generate shorter clauses, sentences, and T-units, less subordination, and fewer noun phrases than NS in college-level writing (Ai and Lu, 2013).

Handling grammatical complexity is a challenging task when two competing aims in academic writing are at stake: explicitness and conciseness (Sawyer et al., 2008; Biber and Gray, 2010). It should be emphasized that the term “explicitness” is borrowed from Mauranen (1993), who claims that an explicit communication is overt and simple, making it easy to comprehend and absorb. On the one hand, sophisticated syntactic structures like nominal phrases in sentences and T-units help academic writers achieve a certain level of conciseness.

In addition, scholars have also identified that academic writing is distinguished by lengthier sentences and T-units, as well as a high number of subordinations and nominalizations (Odonnell, 1974; Brown and Yule, 1983; Hughes, 1996; Halliday 1993; Martin 1993). By minimizing duplication, these grammatical features make the writing more succinct. Complex syntactic structures that are compressed, on the other hand, may diminish the clarity of the intended meaning, which contradicts the objective of explicitness in academic writing. For example, Biber and Gray (2010) suggest that the substantial phrasal complexity, particularly noun-noun phrases, makes the statement of logical relationships between parts in academic writing implicit rather than apparent. Furthermore, complicated grammatical patterns may make research publications difficult to understand (Rottensteiner, 2010; Otto et al., 2012; Dolnicar and Chapple, 2015).

However, most research on the growth of L2 complexity and its link with overall L2 competence and L2 development has been cross-sectional in character and has not managed to examine the actual progress of individual learners over time. The few studies that have looked at longitudinal development either spanned a relatively short period of time, had a limited number of data collecting sites, or involved a small number of learners (Larsen-Freeman, 2006; Verspoor et al., 2008; Byrnes et al., 2010; Spoelman and Verspoor, 2010; Polat and Kim, 2014).

In addition, most studies of syntactic complexity have only been carried out in a small number of writing samples by English as a foreign language (hereafter EFL) learner based on a limited set of indices. Paucity of literature investigating syntactic complexity is found in university students’ L2 language production employing a large set of syntactic complexity indices by investigating a considerable amount of corpus data.

Against this background, the present study aims at addressing this disparity by clarifying the syntactic complexity changes that have occurred in the academic writing of Japanese university students (Non-native English learners), including undergraduate, graduate, and PhD students. A substantial number of essays written by Japanese university students and native speakers were investigated with the use of a large set of syntactic complexity measures.

The following questions were investigated:

- What is the influence of SC in the writing of Japanese university students observed across different academic levels?
- What kind of SC differences, if any, are identifiable in the comparative analysis of Japanese university students’ writing and that of native speakers? What is the degree to which these differences instantiate in the writing samples analyzed in this study?

### Corpus of the study

The study uses Nagoya Interlanguage Corpus of English (NICE) developed by Sugiura in 2015. The corpus comprises argumentative essays written by Japanese undergraduate, graduate, and PhD students, letting us observe the changes of syntactic complexity from a pseudo-developmental perspective. They were tasked to write on the given topic in 1 h without consulting dictionaries.

The NICE also provides the sub-corpus of essays written by native English-speaking writers. Therefore, the essays were also compared to essays written by natives. Data in Nagoya Interlanguage Corpus of English was not collected under perfect uniform conditions. Some participants were asked to choose topics that they favored from a topic list including 11 social issues (e.g., divorce, suicide, and crime), whereas some were not allowed the freedom and could only write to the ones chosen by supervisors. Meanwhile, proportions of the essays collected from each grade were not balanced, and essays written by the first-year students at university accounted for the largest proportion. However, the ones examined were not influenced by the topic or amount of the essays significantly. Also, measures have been taken to eliminate the negative influence when deemed necessary. The overall distribution of the data is given in the Table 1, 2.

**Table 1:** Sample distribution in NICE.

Grade	NNS							NS		
	U1	U2	U3	U4	M1	M2	D	U	M	D
Number of texts	108	85	22	28	49	32	15	113	63	34
Average length	307.13	294.71	337.82	393.71	379.77	434.53	385.60	621.67	571.53	546.32
SD of length	99.81	106.04	122.58	145.76	130.26	106.33	145.75	181.58	73.11	54.93
Words in total	33,171	25,051	7,432	11,024	18,609	13,905	5,784	70,249	36,007	18,575

“NNS” stands for “Non-native English speakers (Japanese L2 learners of English),” “NS” stands for “Native English speakers,” “U” stands for “Undergraduate students,” “G” stands for “Graduate students,” “M” stands for “Graduate students” “D” stands for “Doctoral students,” numbers stand for different grades.

**Table 2:** Types, tokens, and Guiraud index in essays by grade.

Grade	NNS			NS		
	Type	Token	Guiraud	Type	Token	Guiraud
U1	1,741	33,171	9.56	5,168	70,249	19.5
U2	1,636	25,051	10.34			
U3	927	7,432	10.75			
U4	1,238	11,024	11.79			
M1	1,751	18,609	12.84	3,551	36,007	18.71
M2	1,480	13,905	12.55			
D	1,052	5,784	13.83	2,534	18,575	18.6

## Research method

Studies on syntactic complexity have grown tremendously over the last decades, spawning various kinds of assessment rubrics. Measures such as T-unit complexity ratio (number of clauses per T-units), the dependent clause per clause ratio (number of dependent clauses per clauses), and the dependent clause per T-unit ratio (number of dependent clauses per clauses) were considered to be effective measures of syntactic complexity (Wolfe-Quintero et al.,1998, in Knoch, 2009). Incorporating as many effective indices as possible may lead to higher reliability in the evaluation of syntactic complexity. Therefore, we used 14 measures of syntactic complexity by L2 Syntactic Complexity Analyzer (Lu, 2010).

The system embedded in L2 Syntactic Complexity Analyzer enables automatic analysis of L2 written production, producing 14 indices of syntactic complexity based on the 14 measures shown in Table 3 below. Among those measures, six of them were chosen from the large set of measures which have been reviewed in research synthesis studies by Wolfe-Quintero et al. (1998) and Ortega (2003), another five measures were selected as they have been shown by at least one previous study to have at least a weak correlation with proficiency. In addition, three other measures that have not been explored in previous studies but recommended by Wolfe-Quintero et al. (1998) to pursue further have also been selected. Third-party tools, Stanford parser and Tregex, are involved in this system to analyze the syntactic structure of every sentence and calculate the appearance of different kinds of units and syntactic structures.

**Table 3:** A description of indices in L2 Syntactic Complexity Analyzer.

Abbreviation	Index name	Index definition
MLC	Mean length of clause	Number of words/number of clauses
MLS	Mean length of sentence	Number of words/number of sentences
MLT	Mean length of T-unit	Number of words/numbers of T-units
C/S	Sentence complexity ratio	Number of clauses/number of sentences
C/T	T-unit complexity ratio	Number of clauses/numbers of T-units
CT/T	Complex T-unit ratio	Numbers of complex T-units/numbers of T-units
DC/C	Dependent clause ratio	Number of dependent clauses/numbers of clauses
DC/T	Dependent clauses per T-unit	Number of dependent clauses/numbers of T-units
CP/C	Coordinate phrases per clause	Number of coordinate phrases/numbers of clauses
CP/T	Coordinate phrases per T-unit	Number of coordinate phrases/numbers of T-units
T/S	Sentence coordination ratio	Numbers of T-units/number of sentences
CN/C	Complex nominals per clause	Numbers of complex nominals/number of clauses
CN/T	Complex nominals per T-unit	Numbers of complex nominals/numbers of T-units
VP/T	Verb phrases per T-unit	Number of verb phrases/number of T-units

Based on data from Lu (2010).

## Results and discussions

This part elaborates on the findings and discussion of the two study questions already established in “Results and discussions”.

### Changes in syntactic complexity by different levels

**Figure 1:**
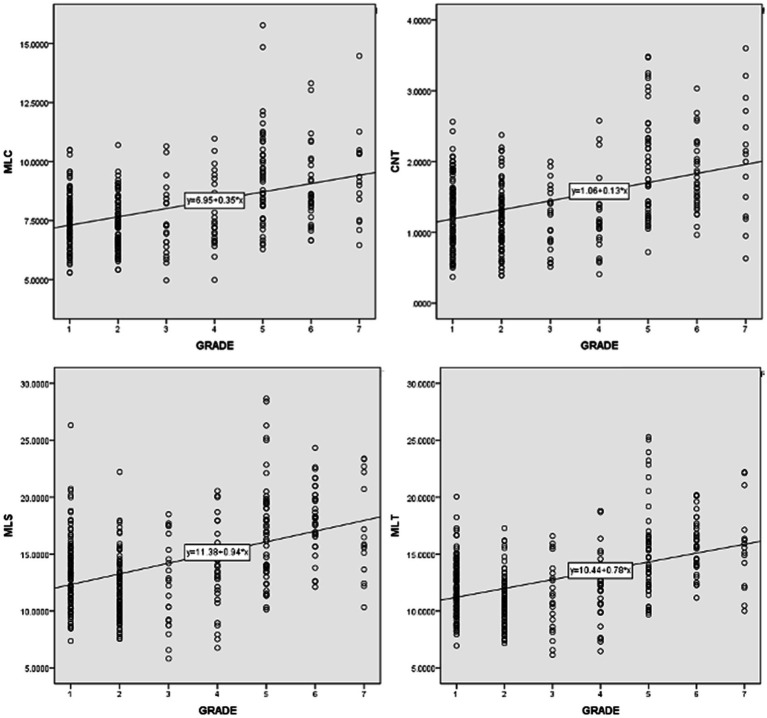
Scatter diagrams of indices which moderately correlate with grades.

**Figure 2:**
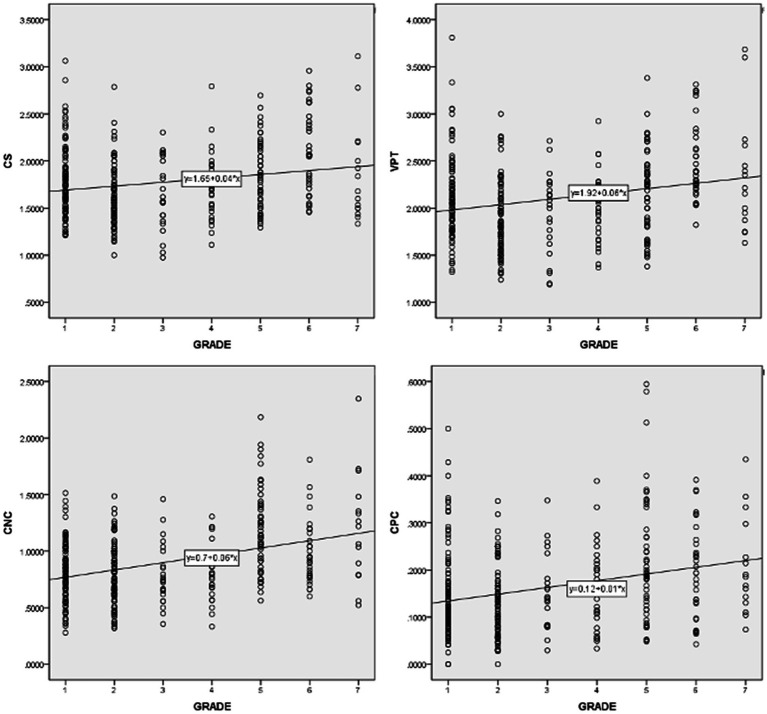
Scatter diagrams of indices which have weak correlations with grade.

Pearson correlation coefficient were computed to assess the relationship between learners’ levels and the 14 syntactic complexity scores of their essays *via* SPSS. Table 4 shows the correlations among individual syntactic complexity indices and grade. It was found that learners’ grades and all the 14 syntactic complexity indices of Japanese learners’ essays were positively correlated. A correlation coefficient ranges from 0.6 to 1.0 indicates a strong linear relationship between variables, 0.4 to 0.6 a moderate correlation, 0.2 to 0.4 a weak correlation, and 0.0 to 0.2 negligible or no correlation. Accordingly, indices such as MLS [*r*(337) = 0.446, *p* < 0.01], MLT[*r*(337) = 0.447, *p* < 0.01], MLC[*r*(337) = 0.428, *p* < 0.01], and CN/T[*r*(337) = 0.407, *p* < 0.01] show moderate correlations with grade. And indices of C/S [*r*(337) = 0.205, *p* < 0.01], VP/T [*r*(337) = 0.240, *p* < 0.01] and CP/C [*r*(337) = 0.274, *p* < 0.01] indicate weak correlations as grade goes up. Scatter diagrams in Figures 1, 2 are those indices which have moderate and weak correlations to grades. According to these results, a larger proportion of Japanese university students in higher grade tend to produce writings of high syntactic complexity. Another important finding is that other indices, namely C/T [*r*(337) = 0.176, *p* < 0.01)], DC/C [*r*(337) = 0.140, *p* < 0.01)], DC/T [*r*(337) = 0.166, *p* < 0.01)], T/S [*r*(337) = 0.122, *p* < 0.01)], CT/T [*r*(337) = 0.143, *p* < 0.01)], demonstrate nearly no linear relationship between grades. A possible explanation for these results may be that even syntactic knowledge of Japanese university learners in higher grade is rather limited and cannot properly utilize complicated syntactic structures such as dependent clauses and complex T-units.

**Table 4:** Pearson’s correlation between grades and syntactic complexity indices.

Variables	2. MLS	3. MLT	4. MLC	5. C/S	6. VP/T	7. C/T	8. DC/C	9. DC/T	10. T/S	11. CT/T	12. CP/T	13. CP/C	14. CN/T	15. CN/C
1. Grade	0.446^**^	0.447^**^	0.428^**^	0.205^**^	0.240^**^	0.176^**^	0.140^**^	0.166^**^	0.122^*^	0.143^**^	0.317^**^	0.274^**^	0.407^**^	0.382^**^
2. MLS		0.938^**^	0.652^**^	0.733^**^	0.758^**^	0.642^**^	0.596^**^	0.616^**^	0.436^**^	0.597^**^	0.543^**^	0.373^**^	0.788^**^	0.598^**^
3. MLT			0.719^**^	0.598^**^	0.774^**^	0.659^**^	0.619^**^	0.641^**^	0.107^*^	0.578^**^	0.607^**^	0.430^**^	0.873^**^	0.686^**^
4. MLC				−0.021	0.273^**^	−0.035	−0.016	−0.037	0.026	−0.045	0.576^**^	0.604^**^	0.655^**^	0.803^**^
5. C/S					0.775^**^	0.896^**^	0.816^**^	0.866^**^	0.552^**^	0.841^**^	0.186^**^	−0.050	0.457^**^	0.084
6. VP/T						0.834^**^	0.769^**^	0.817^**^	0.173^**^	0.741^**^	0.319^**^	0.109^*^	0.592^**^	0.272^**^
7. C/T							0.921^**^	0.980^**^	0.131^*^	0.893^**^	0.232^**^	−0.036	0.550^**^	0.135^*^
8. DC/C								0.964^**^	0.108^*^	0.921^**^	0.209^**^	−0.039	0.538^**^	0.169^**^
9. DC/T									0.103	0.909^**^	0.209^**^	−0.050	0.551^**^	0.146^**^
10. T/S										0.212^**^	−0.007	−0.036	−0.003	−0.060
11. CT/T											0.186^**^	−0.053	0.495^**^	0.131^*^
12. CP/T												0.947^**^	0.464^**^	0.414^**^
13. CP/C													0.315^**^	0.386^**^
14. CN/T														0.891^**^
15. CN/C														1

^**^p < 0.01 and ^*^p < 0.05. The value of p shows that significant level of correlation which is necessary to be shown in the table.

### The gap of syntactic complexity among Japanese learners and native speakers

Within SLA research, Linguistic (or structural) complexity, and its subcomponent syntactic complexity, have been emphasized a great deal. And this is perhaps because of a variety of reasons and owes to a variety of theoretical perspectives, employing various methodological approaches (Ortega, 2003; Bulté and Housen, 2018). Linguistic complexity, in most of the cases, seems to have been measured as a dependent variable in L2 research designs, where it has been used as an indicator of L2 performance and L2 proficiency or as an index of L2 development. Ortega (2003) traces a considerable number of authentic empirical evidence in the SLA literature and further confirms a strong association between the (syntactic) complexity of learners’ L2 production and their overall level of L2 development and/or L2.

To explore whether Japanese university students’ use of syntactic structures approaches that of native speakers, an independent-sample t-test was conducted to compare the syntactic complexity indices of the essays written by native speakers and Japanese university students *via* SPSS. Results are presented in Table 5.

**Table 5:** Independent sample test.

Variables	MLS	MLT	MLC	C/S	VP/T	C/T	DC/C	DC/T	T/S	CT/T	CP/T	CP/C	CN/T	CN/C
*T*	−15.947	−15.663	−13.442	−9.239	−9.293	−8.805	−8.297	−8.744	−3.849	−6.758	−11.713	−8.731	−11.128	−8.270
*df*	547	547	547	547	547	547	547	547	547	547	547	547	547	547
Value of *p*	0.000	0.000	0.000	0.000	0.000	0.000	0.000	0.000	0.000	0.000	0.000	0.000	0.000	0.000

According to the results, all syntactic complexity values of Japanese learners’ written production are significantly lower than that of native speakers’ essays, i.e., there are gaps concerning every aspect of syntactic complexity among Japanese learners and native speakers.

A further step of statistical calculation was made to gauge how significant the gap between native and Japanese university students is concerning their syntactic knowledge. The percentage of difference is obtained when dividing native speakers’ syntactic complexity value by its corresponding mean difference. For instance, the percentage of difference in terms of TS is −3%, implying that students’ T/S value is 3% lower than that of native speakers in average. We can therefore have a basic understanding concerning how huge the gap is even though mean can be misleading occasionally, and the data of this very nature needs to be interpreted with caution. A meticulous approach, in this connection, is recommended. The results suggest that gap exists between the writing of learners and native speakers in terms of all indicators. The subject study, in all the above-mentioned stated dimensions, is an endeavor to fill this gap and chart down a roadmap for future research.

The gap in the first few indicators is relatively small, while gap in the last few indicators is significantly huge (Table 6).

**Table 6:** Percentage of differences.

Grade	Mean difference	Value of native speakers	Percentage of differences (%)
T/S	−0.0363307	1.145719	−3
C/T	−0.2636488	1.857302	−14
VP/T	−0.4158671	2.506286	−17
C/S	−0.3609946	2.133125	−17
DC/C	−0.0782718	0.420855	−19
CT/T	−0.1000529	0.536758	−19
MLC	−1.8834714	9.873938	−19
CN/C	−0.2506051	1.145445	−22
DC/T	−0.2485603	0.823141	−30
MLT	−5.6449353	18.362660	−31
CP/C	−0.0759919	0.238088	−32
MLS	−6.8765018	21.023880	−33
CN/T	−0.7117902	2.151029	−33
CP/T	−0.1821987	0.439451	−41

Taken together, two third of the syntactic complexity indices of Japanese university students’ writing production is proved to moderately or weakly correlate with students’ grades. About 40% of students in higher grades can use more complicated structures such as MLT, MLS, MLC, and CN/T than those in lower grades; an even smaller part of Japanese university students slightly improved in CS, VPT, CPC, and CNC. Meanwhile, the syntactic complexity level of learners’ writing production is found to be significantly lower than that of native speakers. Syntactic structures such as CN/C, DC/T, MLT, CP/C, MLS, CN/T and CP/T seem to be rather difficult for students to utilize.

A significant number of studies, keeping primary research foci into consideration were conducted in pursuit of L2 complexity and its development with the passage of time; usage-based theories of SLA or those adopting a dynamic systems theory (DST) approach are some of the examples (Larsen-Freeman, 2006; Verspoor et al., 2008; Spoelman and Verspoor, 2010; Vyatkina et al., 2015). These studies clearly reflect that although the complexity of L2 learners’ performance increases along with their overall L2 proficiency with the passage of time and during the process of L2 development, linguistic complexity and proficiency are witnessed to be not always on the increase in parallel. Also, the increase in complexity is neither linear, constant nor guaranteed for all layers (lexical, morphological, and syntactic) and sub-dimensions (e.g., diversity, compositionality, and sophistication) of linguistic complexity. Instead, following most instances of natural development, it is characterized by variability and change (Lowie and Verspoor, 2015). It is also important to mention that the significant bondage between different dimensions and layers of complexity can be both supportive and competitive (complexity trade-offs), and their correlation can change as the time proceeds. High degree of variability between individual learners and the non-linearity or temporal variation of their individual developmental trajectories were also the outcome of some of the studies. Keeping this very perspective into consideration, the entire process of second language development, and L2 complexity development, is a dynamic process, at times, found to be proceeding gradually, but with sudden spurs in some cases. Some of its other attributes comprised stages of backsliding and stagnation too. Despite emergence of general developmental patterns and trends across learners (i.e., in groups of learners), the idea of “the average learner” could not be witnessed. However, it is pertinent to mention that the developmental pathways of individual learners need to be investigated to establish a connection with the developmental process.

## Discussions

This section discusses the comparative data and addresses the research objectives stated earlier. Since the study is directed to investigate variations observed in syntactic complexity development, we expected the learners’ overall L2 proficiency, including their knowledge and mastery of syntax, to increase over time, given the significant accumulative amount of L2 English input that the learners in this study were exposed to, as well as the length of exposure each one of them possessed. The rationale why Japanese language learners lag in productive syntactic competence and why the syntactic complexity indices witness different level of improvement is given as under which is in complete consonance with research questions stated above:

### Language learning environment

The restricted response of target language may be the primary barrier which hinders the development of Japanese university students’ syntactic competence. One of the major differences which distinguishes English Taught as a Second Language (ESL) and English Taught as a Foreign Language (EFL) is that leaners in EFL environment receive quite a limited exposure to the target language, let alone the opportunity to interact with native speaker (Lightbown et al., 1993). Students learning English in Japan, a typical EFL environment, are hardly exposed to proper English settings They may experience no difficulty in understanding the varied grammatical structures, but barely use them when speaking or writing English. Therefore, the syntactic complexity in their writing has not grown significantly even they have been learning English for many years. Meanwhile, university students not planning to work or study overseas may lack motivation to advance their English proficiency. As mentioned above, participants majored in quite diversified fields, and most of them are non-English majors. Possibly only those with higher motivation kept learning English and achieved relatively notable improvement in syntactic competence, whereas the others’ language proficiency has stalled.

### Cognitive complexity

Among the 14 syntactic complexity indices, the gap between Japanese university students and native speakers is different. Some of them witnessed narrow gaps between students’ performance and that of native speakers, whereas the others showed significantly huge gap. It is possible to hypothesize that these differences here are likely to be contributed by the different cognitive complexity level of each syntactic structure. For instance, the grammar of complex clauses, such as subordination, is not frequently used in natural language and relatively difficult to process (Givón, 2009). Those marked clause-types may require extensive cognitive processing load.

Conclusively, when syntactic complexity researchers look beyond structural and formal approaches, they can investigate functional motivations for syntactic complexity while keeping an eye out for developmental interfaces with semantic, morphological, and discourse-pragmatic areas of the language that are also subject to developmental explanations. This analytical developmental method opens new avenues for research connecting syntactic complexity with lexical complexity and accuracy, which is currently understudied, however, will be worth investigating in the future. It expands the scope of what may be examined along the whole developmental trajectory within a specific sort of complexification method.

## Conclusion

The aim of the present research was to examine the development of Japanese university students’ productive syntactic competence. This study has shown that a relatively small proportion of Japanese university students in higher levels tends to use more complicated and diversified syntactic structures. Moreover, the research has also clarified the significant difference among the use of syntactic structures by native speakers and Japanese university students. As per the findings of this study, the conceptualization of syntactic complexity in L2 writing research comprises a more comprehensive and fine-grained collection of characteristics than those examined in theoretical frameworks for writing evaluation or in L2 writing rating scales, respectively. Moreover, it supremely emphasizes on the sophistication component of syntactic complexity, which is lacking from holistic scales putting forth a new dimension to the existing research on syntactic complexity. It also provides more precise definitions of syntactic complexity, which may be useful in the development of an automated analysis of syntactic complexity.

All the explicitly elaborated modalities of the method and framework and findings completely conforming to the research objectives will be of interest to teachers and textbook compilers in Japan who are all set to incorporating fundamental changes in the teaching methods of English writing. Further studies, examining data from developmental learner corpus, could open new avenues to the developmental pattern of learners’ syntactic competence.

## Data availability statement

The raw data supporting the conclusions of this article will be made available by the authors, without undue reservation.

## Author contributions

All authors listed have made a substantial, direct, and intellectual contribution to the work and approved it for publication.

## Conflict of interest

The authors declare that the research was conducted in the absence of any commercial or financial relationships that could be construed as a potential conflict of interest.

The reviewer SN declared a shared affiliation with the author MC to the handling editor at the time of the review.

## Publisher’s note

All claims expressed in this article are solely those of the authors and do not necessarily represent those of their affiliated organizations, or those of the publisher, the editors and the reviewers. Any product that may be evaluated in this article, or claim that may be made by its manufacturer, is not guaranteed or endorsed by the publisher.
